# The Edinburgh Tuberculosis Decision

**Published:** 1921-04-16

**Authors:** 


### THE EDINBURGH TUBERCULOSIS DECISION.

In the year before the war the Town Council of Edinburgh transferred to its own management the Royal Victoria Hospital for Consumption, the tuberculosis dispensary, with a farm colony attached to these institutions, retaining the services of Sir Robert Philip, M.D., at an annual salary to guide and direct the work. Near the end of last month it approved a recommendation by its Public Health Committee that the present arrangement with Sir Robert Philip and Dr. James, in connection with the city's tuberculosis scheme, be terminated at an early date; the voting was twenty-two to fourteen. Now, as might be expected, the events of which this change is the upshot are of a varied nature. To begin with, the proposer of the recommendation stated that during the war the military authorities took over the hospital, so that the city had no accommodation there for its patients—"yet they had a consulting expert to whom they paid £500 a year." Before the conclusion just mentioned was come to, requests from the University of Edinburgh and from the Royal Victoria Hospital Trust, both asking for delay and reconsideration of the position, were examined and rejected. It was alleged that the positions of these bodies would not be prejudiced by the changes being made.

We agree with our contemporary, the *Scotsman*, that the manner of the alteration is rather abrupt and unfortunate. Sir Robert Philip's great organising ability deserves, as it has hitherto abundantly received, respect and appreciation. But the fact itself does not greatly surprise us. Setting aside the anti-authoritative spirit of the present time, there is no doubt that in lay minds, at any rate, the phenomenon of the huge war increase in tuberculosis has again discredited those high-pitched claims with which, unfortunately, the contagonist tuberculosis school is so ready. Among the men physically picked for war purposes, who had been living away from all infection, tubercle broke out freely—of course by reason of the physical impairment suffered from war hardships. It is now seen that tuberculosis prophylaxis, like every other modern problem, is a thing the complexity of which increases as our experience of it extends and matures. And this only recently adequately recognised factor, the personal constitutional resistance, has some very wide implications indeed. For a simple instance, if we want to abolish tubercle, why then we must have no more war. Also we must have a vast measure of social amelioration, of uplifting of the working class. As a matter of practical politics, the sociological side of anti-tuberculosis work at present largely outweighs in importance the medical aspect. Naturally enough, such arguments will appeal powerfully to the new *personnel* that in strong force now is coming into municipal life. The didactic attitude of the doctor and health visitor of ten years ago, although still possessing value, is being nowadays discredited, and social reform gaining in favour at the expense of hygienic education. We express no opinion whatsoever upon this; but at the same time feel constrained to agree that the proposition that the current tuberculosis dispensary scheme is a sufficient machinery with which to abolish the disease, is absurd.

There is talk of personal feeling in connection with the affair under notice. Of that, too, we are glad to steer a clear track. We simply recall to our readers a truism or two. On the one hand, favour of all kinds, popular favour especially, is proverbially fickle. For the other, Emerson put it well when he said that the hero is apt at last to become a bore. These two are often, perhaps, faces of one truth. There are signs of more cleavage to occur amongst tuberculosis workers, lay and medical. All this is why responsible laymen keep such a keen look-out for the long-awaited specific remedy, often mistaking meteors for the morning star. Again, the anti-tuberculosis campaign has recently fallen into the political procession and followed humbly at its tail, instead of preserving its real international status. That matter, too, requires rectification. There is no need for despondency, however. Controversy is the breath of science. And truth, as Bryant quaintly put it, "survives if you run over her with a locomotive, while error dies of lockjaw if she pricks her little finger."

